# Extent, Type and Reasons for Adaptation and Modification When Scaling-Up an Effective Physical Activity Program: Physical Activity 4 Everyone (PA4E1)

**DOI:** 10.3389/frhs.2021.719194

**Published:** 2021-11-17

**Authors:** Matthew Mclaughlin, Elizabeth Campbell, Rachel Sutherland, Tom McKenzie, Lynda Davies, John Wiggers, Luke Wolfenden

**Affiliations:** ^1^School of Medicine and Public Health, University of Newcastle, Callaghan, NSW, Australia; ^2^Hunter New England Population Health, Wallsend, NSW, Australia; ^3^Hunter Medical Research Institute, New Lambton Heights, NSW, Australia; ^4^Priority Research Centre for Health Behaviour, University of Newcastle, Callaghan, NSW, Australia

**Keywords:** process evaluation, scale-up, fidelity, implementation science, physical activity, modification, adaptation, schools

## Abstract

**Background:** Few studies have described the extent, type and reasons for making changes to a program prior to and during its delivery using a consistent taxonomy. Physical Activity 4 Everyone (PA4E1) is a secondary school physical activity program that was scaled-up for delivery to a greater number of schools. We aimed to describe the extent, type and reasons for changes to the PA4E1 program (the evidence-based physical activity practices, implementation support strategies and evaluation methods) made before its delivery at scale (adaptations) and during its delivery in a scale-up trial (modifications).

**Methods:** The Framework for Reporting Adaptations and Modifications-Enhanced (FRAME) was used to describe *adaptations* (planned and made prior to the scale-up trial) and *modifications* (made during the conduct of the trial). A list of adaptations was generated from a comparison of the efficacy and scale-up trials via published PA4E1 protocols, trial registrations and information provided by trial investigators. Monthly trial team meetings tracked and coded modifications in “real-time” during the conduct of the scale-up trial. The extent, type and reasons for both adaptations and modifications were summarized descriptively.

**Results:** In total, 20 *adaptations* and 20 *modifications* were identified, these were to physical activity practices (*n* = 8; *n* = 3), implementation support strategies (*n* = 6; *n* = 16) and evaluation methods (*n* = 6, *n* = 1), respectively. Few adaptations were “fidelity inconsistent” (*n* = 2), made “unsystematically” (*n* = 1) and proposed to have a “negative” impact on the effectiveness of the program (*n* = 1). Reasons for the adaptations varied. Of the 20 modifications, all were “fidelity consistent” and the majority were made “proactively” (*n* = 12), though most were “unsystematic” (*n* = 18). Fifteen of the modifications were thought to have a “positive” impact on program effectiveness. The main reason for modification was the “available resources” (*n* = 14) of the PA4E1 Implementation Team.

**Conclusions:** Adaptations and modifications to public health programs are common. Modifications have the potential to impact the implementation and effectiveness of programs. Our findings underscore the importance of comprehensive reporting of the extent, type and reasons for modifications as part of process evaluations, as this data may be important to the interpretation of trial findings.

**Clinical Trial Registration:** https://www.anzctr.org.au/Trial/Registration/TrialReview.aspx?id=372870, Identifier ACTRN12617000681358.

## Introduction

Physical activity has extensive benefits for health and society (1, 2). One in four adults and four in five adolescents globally are insufficiently active to meet aerobic physical activity guidelines (3, 4). While there are an abundance of evidence-based programs to address physical activity, many of these have been tested under optimal research conditions (5, 6), and few have successfully targeted adolescent physical activity (7, 8). As such, many programs previously tested have utilized technical expertise, skills, resources and infrastructure that are not common in real-world operational environments where they are intended to be implemented (9, 10). Further, research trials often recruit participant groups that differ markedly from those of the target population (11).

As physical activity programs examined in research trials are often unsuitable for replication in more real-world environments, they are frequently changed by end-users as part of efforts to make them more suitable for implementation and scale-up (9, 12). These changes can take two forms – *adaptations* to a program prior to it being delivered, and *modifications* that occur during the delivery of the program. *Adaptation* (8, 13) has been defined as a process of thoughtful and deliberate alteration to the design or delivery of a program, with the goal of improving its fit or effectiveness in a given context (13). Program adaptations can include both adaptations to the evidence-based practices and/or to the implementation support strategies provided to increase adoption of the practices in the setting (such as training for clinicians or teachers who will be delivering the program). Adaptations include those to core components of the program, cultural adaptations, mode of delivery adaptations, target audience adaptations and service setting adaptations (14). *Modification* has been defined as encompassing any change to a program, whether deliberately and proactively, or in reaction to unanticipated challenges that arise in the context of its delivery (13). Adaptations and modifications can also be made to evaluation methods.

Systematic reviews demonstrate that program adaptations are ubiquitous as part of efforts to scale-up programs in practice. For example, a systematic review of physical activity programs (8) reported that 100% of programs made adaptations to the program tested in an efficacy trial prior to undertaking a trial of its scale-up. The majority of adaptations focused on the “delivery mode” of programs (8, 15, 16), such as giving preference to online or telephone over face-to-face delivery modes, which are often undertaken to enable greater program reach (8). Understanding program adaptations and modifications is important as they can have significant implications to the effectiveness of programs (9, 12, 17). They have been attributed, in part, to a phenomenon labeled “voltage drop” whereby the effects of a program are reduced by 25–50% when they are implemented at scale in real world contexts (8, 16, 17). However, they have also been hypothesized to improve the impact of programs. For example, improvements may be made by allowing tailoring of evidence-based programs and their implementation (i.e., the local culture, historical context, priorities and availability of funding, staffing and resources), strengthening key program components, reducing inequities by improving its cultural relevance, or reducing relative costs via delivery using less expensive modalities (12). Understanding the nature of program adaptations and modifications is also important for the development of explanations about how they may impact program implementation and outcomes as part of trial process evaluations (18).

The Framework for Reporting Adaptations and Modifications-Enhanced (FRAME) (13, 19) was recently developed to support the consistent documentation and reporting of program adaptations and modifications. It provides a taxonomy of classifying adaptations and modifications (13) including *what* is adapted/modified, the *nature* of the adaptation/modification, *who* participated in the adaptation/modification decision, for *whom*/*what* is the adaptation/modification made and *when* it occurred. Despite the existence of FRAME and the need for consistent reporting, both adaptations made prior to program delivery and modifications made during the implementation are often poorly described in research reports (13, 15, 20, 21). That is, individual trials seldom report adaptations for scale-up (prior to program delivery) using consistent taxonomies (13), instead trials rely on descriptions of adaptations that can't be compared between trials (13).

A systematic review of 42 evidence-based public health programs that reported adaptations to the evidence-based program practices (15) found that the most frequent types of adaptation were tailoring (93%) or adding elements (71%). Most commonly these adaptations were to content (100%), context (95%), cultural (74%) and/or delivery (62%). While the review provides useful insights into the frequency of adaptations to the evidence based program practices, it does not explicitly include adaptations to the implementation support strategies used or the evaluation methods (13, 22). Also, the authors relied on published papers to retrospectively code adaptations to evidence-based program practices (15). A limitation of relying on published papers is that sometimes the extent, types, context and reasons for adaptations and modifications may be unclear or absent completely from these documents, remaining instead with those people involved in the scale-up process (15). Additionally, it is also unclear in many programs what modifications occur during delivery, and to the authors' knowledge, no physical activity studies have used a consistent taxonomy to report modifications during program delivery (23). Importantly, prior studies have also not routinely reported who was responsible for program adaptations or modifications, why these were undertaken, and if they were considered to contribute, or detract, from the effects of the program. Such information could be used to help interpret trial findings in implementation-effectiveness studies (13, 22, 24).

In the absence of well-described adaptations prior to delivery and modifications during delivery, we present here a descriptive study of the adaptations and modifications made in the scale-up of an evidence based physical activity program targeting adolescents, Physical Activity 4 Everyone (PA4E1). PA4E1 is a secondary school physical activity program. After an efficacy trial, PA4E1 showed positive results (25–29), PA4E1 was adapted in preparation for scale-up (30). The PA4E1 program includes both an evidence-based program (consisting of seven school physical activity practices) and seven implementation support strategies offered to help schools implement these physical activity practices (implementation support). The aims of the current paper are:

1. To describe the extent, type and reasons for adaptations to PA4E1 that were made for scale-up to the physical activity practices, implementation support strategies and evaluation methods.
2. To describe the extent, type and reasons for modifications during the PA4E1 scale-up trial made to the physical activity practices, implementation support strategies and evaluation methods.

## Methods

This research has been conducted and reported in accordance with the requirements of the Standards for Reporting Implementation Studies (StaRI) Statement (Additional File 1) and Template for Intervention Description and Replication (TIDieR) checklist (Additional File 2).

### Ethical Approval

The efficacy and scale-up trials have been registered at ACTRN12612000382875 and ACTRN12617000681358, respectively. Ethical approvals were sought from Hunter New England Human Research Ethics Committee (Ref No. 11/03/16/4.05), University of Newcastle (Ref No. H-2011-0210), NSW Department of Education and Communities (SERAP 2011111), Maitland Newcastle Catholic School Diocese, Broken Bay Catholic School Diocese, Lismore Catholic School Diocese, Armidale Catholic School Diocese, and the Aboriginal Health and Medical Research Council (AHMRC).

### Stages of Physical Activity 4 Everyone (PA4E1)

**Figure 1:**
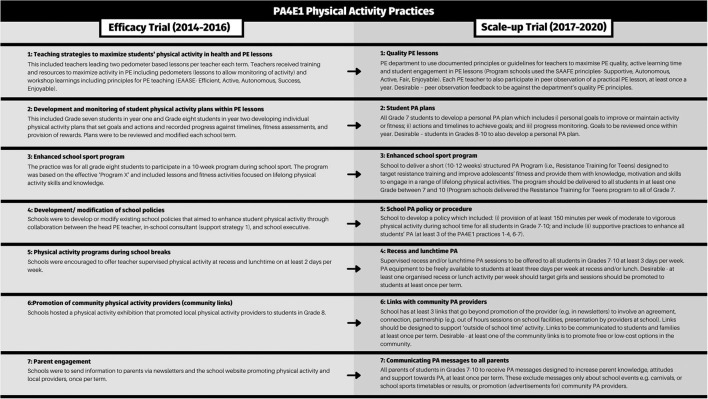
Summary of the physical activity practices in the PA4E1 efficacy trial (25–27, 29) and the PA4E1 scale-up trial (30–32).

**Figure 2:**
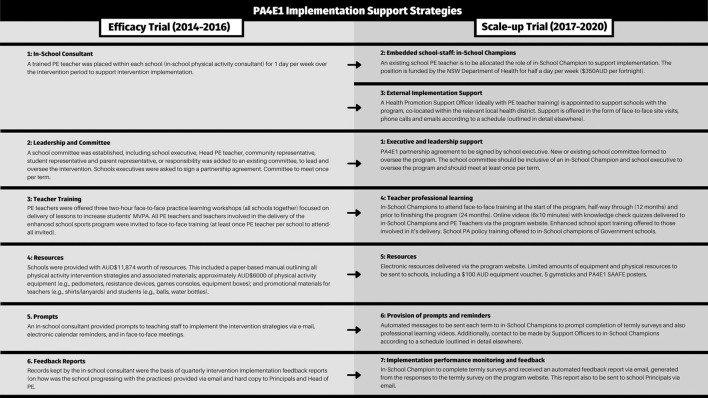
Summary of the implementation support strategies offered to schools in the PA4E1 efficacy trial (25–27, 29) and the PA4E1 scale-up trial (30–32).

An outline of the physical activity practices (evidence based program) for both the efficacy and scale-up trials are shown in Figure 1. The implementation support strategies offered to schools are outlined for both trials in Figure 2 (26, 27, 29–32). As is best practice in implementation science (33), we distinguish between program components, separating the evidence-based program practices (the physical activity practices) from the implementation support strategies, which are designed to assist schools to implement the physical activity practices.

#### Efficacy Trial (2012–2014)

The evaluation methods of the PA4E1 efficacy trial have been reported in a trial protocol (26). Briefly, the PA4E1 efficacy trial was a 2-year (2012–2014) cluster randomized controlled trial involving 10 low-socioeconomic Australian secondary schools (five per group). Hunter New England Local Health District (HNELHD) led PA4E1 (34), supported by two other local health district delivery partners (26, 34) in a research-practice partnership with the University of Newcastle and New South Wales (NSW) Department of Education. PA4E1 had positive effects on students' device-measured moderate-to-vigorous physical activity and unhealthy weight gain (25–27, 29) and was deemed cost-effective (28). The PA4E1 program consisted of seven physical activity practices (Figure 1) to support students to be more physically active (the evidence-based program) and six implementation support strategies designed to embed the physical activity practices within the school environment (Figure 2).

#### Adaptation Process (2017)

Adaptations were made in 2017 with the goal of scaling up the program (physical activity practices and implementation support strategies) employed in the efficacy trial and testing the effects again as part of a scale-up trial. The process has been reported in more detail elsewhere (30). The scale-up adaptation process sought to retain the effects of the original program by retaining components deemed as core (physical activity practices and implementation support strategies) (25–27, 29) while enabling greater reach (scaling up to more schools). Briefly, to adapt PA4E1 for scale-up, we used a four-stage iterative scale-up process, based on a review of existing models and factors for scaling up public health programs (35) and a scoping review of frameworks for adapting public health programs (36). Firstly, we identified barriers and enablers to the physical activity practices and implementation support strategies. Second, we mapped the identified barriers to the Theoretical Domains Framework (37) and the Behavior Change Wheel (38). Thirdly, we prioritized the components of the program from the perspective of a health service requiring its delivery at scale, considering variables such as affordability, practicability and acceptability (39). Finally, the PA4E1 Expert Advisory Group (comprised of senior health service staff, senior academics, NSW Education sector partners and the PA4E1 project staff) reviewed the prioritized program (physical activity practices and implementation strategies) and made the final judgement regarding the design and components of the resulting PA4E1 program (30).

#### Scale-Up Trial (2017–2019)

The scale-up trial was a type III hybrid implementation-effectiveness trial (24). Methods have been reported in both a trial protocol (30) and a process evaluation protocol (32). The scale-up trial (2017–2019) tested the adapted PA4E1 program in a larger number of low-socioeconomic secondary schools (*n* = 49) across a larger geographic area (HNELHD leading three other local health districts, in a research-practice partnership with the University of Newcastle and NSW Department of Education) (34). Program schools were offered seven implementation support strategies (incorporating 23 sub-strategies) to support their adoption of seven physical activity practices (30–32). The scale-up trial recruited 49 schools (24 program, 25 control) (31).

### Defining Adaptation and Modification

We operationally defined changes to PA4E1 (inclusive of the physical activity practices, implementation support strategies and the evaluation methods) temporally, as either *adaptations* which were planned and made prior to the scale-up trial or *modifications* which were made during the conduct of the trial. The methods are reported by aim. Aim one focuses on adaptations (prior to scale) and aim two focuses on modifications (during implementation of the program in the scale-up trial).

### Measures and Procedures

The measures and procedures are reported by aim.

Aim 1: To describe the extent, type and reasons for adaptations to PA4E1 that were made for scale-up to the physical activity practices, implementation support strategies and evaluation methods.

We used the FRAME framework to code the adaptations to the PA4E1 program made for scale-up (13). We applied the FRAME framework coding to the evidence-based practices (physical activity practices), the implementation support strategies (13) and the evaluation methods. The FRAME framework was used to provide a discrete set of codes for each category of adaptation (see **Table 2** for a list of codes for each category). Additional coding categories (outlined below) were developed by the author team in line with methods from Rabin et al., (23) to report free-text descriptions of each adaptation.

**Table 2** shows the adaptation categories utilized and the response codes. Briefly, these are:

- Description of the adaptation (open text)^*^
- What component of the PA4E1 program was the adaptation made to (the physical activity practices, implementation support strategies or evaluation methods)?
- Was the adaptation proposed to have a positive, null or negative impact on the program effectiveness at the time of adaptation?

The following categories were coded for each adaptation from the FRAME framework (6):

- Were adaptations systematic or unsystematic?
- Note: Based on the Model for Adaptation and Impact (MADI) framework (20), we revised the FRAME framework (13) terminology to remove the code “planned” and replace it with the code “systematic.” This emphasizes the importance of how the modification was made (i.e., was it done using a systematic process which involved the use of theory to make the adaptation?). We operationalized the code “systematic” and applied it to adaptations that used both theory and a process to make the adaptation. By contrast, “unsystematic” was the code assigned when either a theory was not used or a process was not used to make the adaptation.
- Were adaptations proactive or reactive?
- Who participated in the decision to adapt? (e.g., program manager, individual practitioners such as in-School Champion)
- What was the goal of adaptation? (e.g., improve feasibility, reduce cost)
- What was adapted? [i.e., context, training and evaluation, implementation and scale-up activity, content (including aspects of the way content was delivered)]
- Context adaptations were made to what? (e.g., format, setting)
- At what level of delivery did adaptations occur? (e.g., unit level - an individual school)
- What is the nature of the content adaptation? (e.g., adding elements, shortening/condensing)
- Were adaptations fidelity consistent (relationship to core elements retained) or fidelity inconsistent?
- Reasons for the adaptation? (including socio-political, organization/setting, provider and recipient reasons).

Certain coding categories were considered “not applicable” to the evaluation methods (as outlined in **Table 2**).

Firstly, MM identified a list of adaptations between the efficacy and scale-up trial on physical activity practices, implementation support strategies and evaluation by triangulating data from a number of sources. The first source was published PA4E1 research papers: the trial protocol (26), 12 and 24-month outcome papers (27, 29) and cost-effectiveness paper for the efficacy trial (28); and the trial protocol (30), process evaluation protocol (32) and 12-month practice outcome paper (31) for the scale-up trial. Secondly, we drew upon the trial registries for both trials. Finally, to provide important context and resolve discrepancies in recorded adaptations, we drew on the historical knowledge of three authors (LD, EC and RS) who were involved in both trials through meetings with the lead author (MM).

To refine the initial codes, a consensus meeting was held between MM, TM, LD, RS, and EC. Following discussion, MM then finalized the coding for each of the adaptations. Finally, the final codes were agreed upon by email by MM, TM, LD, RS, and EC. Descriptive statistics for each adaptation category were calculated (e.g., the number and type of adaptations that were made).

To synthesize the adaptations, we calculated descriptive statistics for each adaptation category (e.g., the number and type of adaptations that were made) overall, and for adaptations made to physical activity practices, to implementation support strategies and to evaluation.

Aim 2: To describe the extent, type and reasons for modifications during the PA4E1 scale-up trial made to the physical activity practices, implementation support strategies and evaluation methods.

We used the FRAME framework to describe and code modifications made during the delivery of the scaled-up PA4E1 program to the physical activity practices, the implementation support strategies and the evaluation methods.

We used the method as outlined in the process evaluation protocol (32). Throughout the 24-month program (2017–2019), Support Officers involved in the delivery of the program to schools and the PA4E1 Management Team involved in the day-to-day operations of the project continually tracked modifications to PA4E1 during the program in “real-time” by adding them to a Microsoft Excel spreadsheet (23). A monthly meeting (up to 60 min) was held between the PA4E1 Management Team (including at least one Support Officer) to code the identified modifications onto the Stirman et al., (13) framework for modifications in the same spreadsheet (19). MM subsequently updated the coding to reflect the additional categories included within the updated framework by Stirman et al., the FRAME framework (13, 19). This method of ‘real-time ongoing coding’ has previously been found to be feasible to track modifications (23). We used the same coding categories from Aim 1 for each modification (replacing the word adaptation for modification), as well as coding two additional categories. For the category of proposed impact on the program effectiveness at the time of modification (i.e., “positive”, “null” or “negative”), we coded this based on the predicted impact of the modification at the time of coding, rather than the actual or measured impact of the modification.

- Reason for the modification (open text)
- School term the modification was first applied (within the nine school term program).

At the end of the program, the final codes were collated by MM. The final codes were discussed and refined (by RS, EC, LD, TM) to reach consensus. Descriptive statistics for each modification category were calculated (e.g., the number and type of modifications that were made) overall and separately for physical activity practices, implementation support strategies and evaluation methods.

## Results

Results are reported by adaptations (aim 1) and modifications (aim 2), respectively.

### Adaptations

Aim 1 was to describe the extent, type and reasons for adaptations to PA4E1 that were made for scale-up to the physical activity practices, implementation support strategies and evaluation methods. Table 1 descriptively summarizes the main program adaptations from efficacy trial to scale-up trial for individual physical activity practices and implementation support strategies, including the codes “fidelity consistent or fidelity inconsistent”, “systematic or unsystematic” and “proposed positive, negative or null impact on the project” (as described in the methods). For a more expanded description of adaptations made for the scale-up trial and their coding, see Additional File 3.

**Table 1:** Adaptations to each specific component of PA4E1 efficacy trial made for the scale-up trial, including adaptations to individual school physical activity practices and implementation support strategies offered to schools.

**Name of efficacy trial program component**	**Following adaptation, name of scale-up trial program component**	**Descriptive summary of main adaptations from efficacy trial to scale-up trial**	Fidelity consistent?^*^	Systematic?^*^	**Proposed positive (+), negative (-) or null (0) impact on the project**
Practice 1: teaching strategies to maximize students' physical activity in health and physical education (PE) lessons	**Practice 1**: quality PE lessons	• Change of focus from focusing on “Active” (which is a single principle within “SAAFE” PE lesson guidelines) to all “SAAFE” principles PE Lessons (i.e., supportive, active, autonomous, fair and enjoyable) (40). • Though pedometers were made available to schools, this became less of a focus and was not a mandatory part of the practice. • The PE Department used documented principles or guidelines. • PE teacher should participate in peer observation of a practical PE lesson at least once per year.	**X**	✓	**+**
Practice 2: development and monitoring of student physical activity plans within PE lessons	**Practice 2**: student PA plans	• As well as Grade 8's in year two, Grade 7's should also develop a physical activity plan in year two. • Goals were to be reviewed yearly, not termly.	✓	✓	**+**
Practice 3: enhanced school sport program	**Practice 3**: enhanced school sport program	• Changed from “Program X” (41) to the “Resistance Training 4 Teens (RT4T)” program (42). • RT4T was offered as accredited training by the NSW Department of Education.	✓	✓	**+**
Practice 4: development/ modification of school policies	**Practice 5**: school PA policy or procedure	• The policy must include the provision of at least 150 min of moderate to vigorous-intensity physical activity during school time for all students in Grade 7–10.	✓	✓	**+**
Practice 5: physical activity programs during school breaks	**Practice 4**: recess and lunchtime PA	• Changed to 3 days per week (from two), ideally with at least one activity targeting girls specifically. • Schools were also asked to provide access to physical activity equipment to students at least 3 days per week.	✓	✓	**+**
Practice 6: promotion of community physical activity providers (community links)	**Practice 6**: links with community PA providers	• Specification that schools form three links with community physical activity providers that go beyond the promotion of the provider. • It was desirable that at least one of the community links made were to promote free or low-cost options in the community. • Schools were asked to use multiple modes to promote (e.g., newsletter, parent app). • This replaced a 1-day community physical activity provider expo (as more feasible and sustained)	✓	✓	**+**
Practice 7: parent engagement	**Practice 7**: communicating PA messages to all parents	• Schools were asked to use multiple modes to communicate the messages (e.g., newsletter, parent app).	✓	✓	**+**
Strategy 1: in-school physical activity consultant	**Strategy 2 and 3**: embedded school staff: in-School Champion and External implementation support	• External physical activity consultant replaced by an in-School Champion (an existing PE teacher within the school) who was supported by a health promotion support officer employed by the respective local health district. • In-School Champions were funded $400 a fortnight. • Support Officer and in-School Champion maintained contact through face-to-face meetings, email and phone according to the schedule documented within the support strategy. • Support Officer was co-located in the same local health district with in-School Champions.	**X**	✓	**+**
Strategy 2: establishing leadership and support	**Strategy 1**: executive and leadership support	• Less total committee members in the scale-up trial (i.e., no requirement for student, parent, Head PE teacher and community representative) and include both the in-School Champion and school executive.	✓	✓	**+**
Strategy 3: teacher training	**Strategy 4**: teacher professional learning	• PE Teacher training *via* a website (six modules) rather than three face-to-face sessions. • NESA (New South Wales Education Standards Authority) accreditation attached to online training. • Specific training for writing a physical activity policy for in-school champions. • Three days training for in-school champion.	✓	✓	**+**
Strategy 4: resources	**Strategy 5**: resources	• Paper-based resources were replaced by a website housing documentation and resources, except for printed posters outlining the SAAFE principles (for practice 1). • Less total equipment provided, five gymsticks and an equipment voucher were provided to schools rather than providing all the $6,000 equipment. • Promotional materials were not issued as part of the support strategy but instead for the completion of evaluation measures.	✓	✓	-
Strategy 5: prompts	**Strategy 6**: provision of prompts and reminders	• Support officers reminded in-school champions to implement the program rather than the in-school consultant reminding teachers. • Automated prompts to in-School Champions and PE teachers delivered *via* the program website.	✓	✓	**+**
Strategy 6: intervention implementation performance	**Strategy 7**: implementation performance monitoring and feedback	• Feedback was automated via the program website and was directly against the physical activity practice milestones (as practice implementation builds over two school years and is designed to be ongoing). • Feedback was automatically sent to (website registered) in-School Champions and Principals. • No direct observations were undertaken by the Support Officer.	✓	✓	**0**

*A total of 20 adaptations were made to scale-up PA4E1, including seven to individual physical activity practices and six to implementation support strategies. A further six adaptations were made to the evaluation methods and one adaptation to all physical activity practices, not included in this table. For a description of each coding category, see the methods and FRAME (13). See Additional File 3 for a complete dataset of the types and reasons for each modification according to the FRAME (13)*.

* *Tick (✓) indicates agreement with the question (i.e., yes). Cross (**X**) indicates disagreement with the question (i.e., no)*.

*PE, Physical Education; SAAFE, Supportive Active Autonomous Fair Enjoyable; RT4T, Resistance Training 4 Teens; NSW, New South Wales.; NESA, NSW Education Standards Authority*.

### Number of Adaptations

A total of 20 adaptations were made to scale-up PA4E1. Eight adaptations were to the school physical activity practices, six to the implementation support strategies and the remaining adaptations were to the evaluation methods (*n* = 6). Table 2 summarizes the codes according to the FRAME framework for physical activity practices, implementation support strategies and evaluation methods, respectively (13). Additional File 3 outlines each individual adaptation in detail and includes the full set of codes outlined in the methods.

**Table 2:** FRAME framework (13) modification codes for adaptations from efficacy to scale-up trial.

**Modification categories**	**Code**	**Physical activity practices** ***n*** **(%)**	**Implementation support strategies** ***n*** **(%)**	**Evaluation methods** ***n*** **(%)**	**Total (practices, strategies and evaluation)** ***n*** (%)^**^
Program component?	Physical activity practices implementation support strategies evaluation	8 (100) N/A N/A	N/A 6 (100) N/A	N/A N/A 6 (100)	8 (40) 6 (30) 6 (30)
Proposed impact on the project?	Positive Negative Null Not applicable	8 (100) 0 (0) 0 (0) 0 (0)	4 (66) 1 (17) 1 (17) 0 (0)	N/A N/A N/A 6 (100)	12 (86) 1 (7) 1 (7) 6^*^
Relationship to fidelity/core elements?	Fidelity consistent Fidelity inconsistent Not applicable	7 (88) 1 (13) 0 (0)	5 (83) 1 (17) 0 (0)	N/A N/A 6 (100)	12 (86) 2 (14) 6
Were adaptations systematic or unsystematic?	Systematic Unsystematic	7 (88) 1 (13)	6 (100) 0 (0)	6 (100) 0 (0)	19 (95) 1 (5)
Were adaptations proactive or reactive?	Proactive Reactive	8 (100) 0 (0)	6 (100) 0 (0)	6 (100) 0 (0)	20 (100) 0 (0)
Who participated in the decision to modify?^*^	Program manager Treatment/Intervention team Other codes	8 (100) 8 (100) 0 (0)	6 (100) 6 (100) 0 (0)	6 (100) 6 (100) 0 (0)	20 (100) 20 (100) 0 (0)
What was the goal?^*^	Improve fit with recipients Improve feasibility Improve effectiveness/outcomes Reduce cost Increase reach or engagement Increase satisfaction Other codes Not applicable	4 (50) 0 (0) 3 (38) 0 (0) 1 (13) 1 (13) 0 (0) 0 (0)	4 (67) 5 (83) 1 (17) 3 (50) 0 (0) 0 (0) 0 (0) 0 (0)	0 (0) 0 (0) 0 (0) 0 (0) 0 (0) 0 (0) 0 (0) 6 (100)	8 (57) 5 (36) 4 (29) 3 (21) 1 (7) 1 (7) 0 (0) 6
What is adapted?^*^	Content Implementation and scale-up activities Training and evaluation Contextual	7 (88) 0 (0) 0 (0) 1 (13)	6 (100) 6 (100) 0 (0) 0 (0)	0 (0) 6 (100) 6 (100) 0 (0)	13 (65) 12 (60) 6 (30) 1 (5)
Contextual modifications are made to what?	Format Setting Personnel Population Not applicable	1 (13) 0 (0) 0 (0) 0 (0) 7 (88)	0 (0) 0 (0) 0 (0) 0 (0) 6 (100)	N/A N/A N/A N/A 6 (100)	1 (100) 0 (0) 0 (0) 0 (0) 19
At what level of delivery were adaptations made?	Target intervention group (all schools) Cohort (group of schools sharing a characteristic) Clinic unit/level (individual schools)	8 (100) 0 (0) 0 (0)	6 (100) 0 (0) 0 (0)	6 (100) 0 (0) 0 (0)	20 (100) 0 (0) 0 (0)
What is the nature of the content adaptation?^*^	Substituting Tailoring/tweaking/refining Lengthening/extending elements Adding elements Shortening/condensing Not applicable	3 (38) 0 (0) 2 (25) 1 (13) 1 (13) 1 (13)	4 (67) 0 (0) 0 (0) 1 (17) 2 (33) 0 (0)	0 (0) 0 (0) 0 (0) 0 (0) 0 (0) 6 (100)	8 (62) 3 (23) 2 (15) 2 (15) 3 (23) 7
Reasons–socio-political (i.e., broad context)^*^	None (no reason) Funding or resource availability/allocation Existing policies Societal/cultural norms Other codes Not applicable	4 (50) 2 (25) 2 (25) 0 (0) 0 (0) 0 (0)	5 (83) 1 (17) 0 (0) 0 (0) 0 (0) 0 (0)	0 (0) 0 (0) 0 (0) 0 (0) 0 (0) 6 (100)	9 (64) 3 (21) 1 (7) 1 (7) 0 (0) 6
Reasons –organization/setting (i.e., PA4E1 Implementation Team)^*^	None (no reason) Available resources (funds, staff, tech, space) Other codes Not applicable	8 (100) 0 (0) 0 (0) 0 (0)	0 (0) 6 (100) 0 (0) 0 (0)	0 (0) 0 (0) 0 (0) 6 (100)	8 (57) 6 (43) 0 (0) 6
Reasons – provider (i.e., Local Health District)^*^	None (no reason) Not applicable	8 (100) 0 (0)	6 (100) 0 (0)	0 (0) 6 (100)	14 (100) 6
Reasons – recipient (i.e., schools and in-School Champions)^*^	None (no reason) Cultural or religious norms Physical capacity Motivation and readiness Access to resources Other codes Not applicable	4 (50) 0 (0) 2 (25) 1 (13) 2 (25) 0 (0) 0 (0)	2 (33) 4 (66) 0 (0) 1 (17) 0 (0) 0 (0) 0 (0)	0 (0) 0 (0) 0 (0) 0 (0) 0 (0) 0 (0) 6 (100)	6 (43) 4 (29) 2 (14) 2 (14) 2 (14) 0 (0) 6

* *Percentages may not sum to 100%, and n values may not add up to the number of adaptations (N = 20) because multiple codes within this category may be applied to each adaptation*.

** *“Not applicable (N/A)” category not included in denominator to calculate percentages*.

*Not applicable, (N/A)*.

*Additional file 3 outlines each individual adaptation in detail and includes the full set of codes as outlined in the methods*.

The vast majority of adaptations were coded as “systematic” (*n* = 19), as they were made during the theory-informed iterative scale-up process. By definition, all adaptations (*n* = 20) were made “proactively” rather than in response to an unknown event or circumstance. Two adaptations were deemed “fidelity inconsistent”, as the core elements or functions had changed as a result of the adaptation (13). Of the 14 adaptations to the practices and strategies, 12 were proposed to have a positive impact on program effectiveness. All adaptations involved both the “Program Manager” and “Treatment/Intervention Team” in the decision-making process, which included the PA4E1 Implementation Team (inclusive of the program manager, project staff and the expert advisory group) as described in the methods.

### Types of Adaptations

The goals and types of adaptations varied. The most common goals were to “improve fit with recipients” (*n* = 8), “improve feasibility” (*n* = 5), “improve effectiveness/outcomes” (*n* = 4) and to “reduce cost” (*n* = 3). Most adaptations were to “content” (*n* = 13) and “implementation and scale-up activities” (*n* = 12). Fewer adaptations were to “training and evaluation” (*n* = 6) or “contextual” (*n* = 1). All adaptations (*n* = 20) were made at the “target intervention group” level, meaning that adaptations applied to all schools receiving the program, rather than certain schools or local health districts (e.g., “individual”, “cohort” or “individual practitioner” level). Content adaptations varied, including “substituting” (*n* = 8) “tailoring/tweaking/refining” (*n* = 3) and “shortening/condensing” (*n* = 3).

### Reasons for Adaptations

The reasons for adaptations included the broad context of having “funding or resource availability/allocation” (*n* = 3) and also the PA4E1 Implementation Team having “available resources (funds, staff, technology, space)” (*n* = 6). School and in-School Champions (i.e., “recipients”) reasons included cultural or religious norms (*n* = 4), “physical capacity” (*n* = 2), “motivation and readiness” (*n* = 2) and “access to resources” (*n* = 2).

### Modifications

Aim 2 was to describe the extent, type and reasons for modifications during the PA4E1 scale-up trial made to the physical activity practices, implementation support strategies and evaluation methods. Table 3 provides a brief description of each modification made to the physical activity practices and implementation support strategies during the scale-up trial delivery.

**Table 3:** Modifications to each component of PA4E1 scale-up trial made during the delivery of the program (practices and implementation support strategies).

**Modification number (term initiated)**	**Program component(s) and brief description of modification made during scale-up trial**	Fidelity consistent?^*^	systematic?^*^	Proactive modification?^*^	**Proposed positive (+), negative (–) or null (0) impact on the project?**
1 (1)	•**Implementation strategy 2:** for the entire duration of the program, funding for the in-school champions was increased from AUD$350 a fortnight to AUD$400.	✓	**X**	✓	**+**
2 (1)	•**Implementation strategy 5:** instead of simply providing the physical resources to schools, they were issued as 'incentives' upon completion of training, though all schools ended up receiving the resources as they all completed the necessary training. All schools ended up receiving the resources if they wanted them.	✓	**X**	✓	**+**
3 (1)	•**Implementation strategy 3:** due to staff turnover, for some schools, their support officer was not co-located within same local health district.	✓	**X**	**X**	**–**
4 (1)	•**Implementation strategy 3:** due to staff turnover, for some schools, their Support Officer not trained in physical education teaching.	✓	**X**	**X**	**–**
5 (2)	•**Implementation strategy 6:** prompting emails were supposed to be sent reminding users to complete professional development. However, these were not sent to PE Teachers or in-School Champions if they registered after the first term of the program. An error in the website coding.	✓	**X**	**X**	**–**
6 (4)	•**Implementation strategy 5:** additional resources were made available, these were a set of 30 pedometers made available to schools who wanted them. Not all schools wanted them. The PA4E1 had three sets available in total.	✓	**X**	✓	**+**
7 (4)	•**Implementation strategy 3:** enhanced school sport training delivered by support officers to a single school as department of education training dates had expired. In-school champions and PE teachers unable to receive accreditation for this *ad-hoc* training.	✓	**X**	**X**	**+**
8 (4)	•**Implementation strategy 4:** extra day of face-to-face training held halfway through the program (Term 6). While a second day of face-to-face training was outlined within the study protocol [Table 2 (30)]. School Champions were not made aware of this until Term 4 of the program. This was because the program team were unsure about available resources.	✓	**X**	✓	**+**
9 (5)	•**Implementation strategy 5:** Facebook group created by in-school champions to facilitate resource, discussion and knowledge exchange.	✓	**X**	✓	**+**
10 (5)	•**Practice 2:** physical activity plans to be completed by Grade 7 only in the second half of the program, not both Grade 7 and 8 [as originally described in the study protocol – see Table 2 (30)].	✓	**X**	✓	**–**
11 (5)	•**Implementation strategy 4:** face-to-face training was repeated for schools unable to attend the centralized training held for all schools. This training was delivered locally to the schools at a location and time that suited the schools, to reduce travel times for the in-school champions.	✓	**X**	**X**	**+**
12 (6)	•**Implementation strategy 5:** additional physical resources for enhanced school sport training were sent to a single school who requested them from their support officer.	✓	**X**	**X**	**+**
13 (6)	•**Practice 1:** lesson observation forms could be submitted either through the website form or uploaded as a word document (new).	✓	**X**	✓	**+**
14 (7)	•**Implementation strategy 7:** all schools were sent incorrect termly survey feedback reports due to an error with the termly survey. A replacement report was sent with the correct
15 (7)	•**Practice 6:** termly survey definition of meeting practice 6, changed from mandatory to desirable to have a low or no-cost option community link.	✓	**X**	✓	**+**
16 (7)	•**Implementation support strategy 1–7:** extension of the whole implementation support program by one school term, extending the program from eight school terms to nine school terms. However, schools were not provided additional funds for release of the in-school champion (Implementation Strategy 2)	✓	✓	✓	**+**
17 (7)	•**Implementation strategy 4:** Extra day of face-to-face training held at the end of the program to support sustainability (Term 9).	✓	**X**	✓	**+**
18 (8)	•**Implementation strategy 7:** sustainability reports, similar to termly surveys and feedback reports, were designed to assist schools to plan strategies for sustaining the PA4E1 program in their school beyond the life of the research project. These were issued via email to be completed by in-School Champions in liaison with their school Principal.	✓	✓	✓	**+**
19 (8)	•**Implementation strategy 1 and 7:** all School Principals were offered a face-to-face meeting in Term 8 to explain their schools 24 month sustainability report.	✓	**X**	✓	**+**

*A total of 20 modifications were made during the scale-up of PA4E1, including three to physical activity practices and 16 to implementation support strategies. A further adaptation was made to the evaluation methods, not included in this table. Term initiated refers to the School Term (1–9) that the modification was first made. For a description of each coding category, see the methods section and FRAME (13). See Additional File 4 for a complete dataset of the types and reasons for each modification according to the FRAME (13). PE, Physical Education; NSW, New South Wales*.

* *Tick (✓) indicates agreement with the question (i.e., yes). Cross (**X**) indicates disagreement with the question (i.e., no)*.

### Number of Modifications

A total of 20 modifications were made during the delivery of the scale-up trial of PA4E1 from 2017–2019. Of these, 16 modifications were made to the implementation support strategies, three to the physical activity practices and one to the evaluation methods. All modifications were deemed “fidelity consistent” and most modifications were proposed to have a positive impact on the effectiveness of the program (*n* = 15). Table 4 summarizes the modification codes according to the FRAME framework (13). Additional File 4 outlines each individual modification in detail and includes the full set of codes (as outlined in the methods).

**Table 4:** FRAME framework modification codes for modifications during the scale-up trial.

**Modification categories**	**Codes**	**Physical activity practices** ***n*** **(%)**	**Implementation support strategies** ***n*** **(%)**	**Evaluation methods** ***N*** **(%)**	**Total (practices, strategies, whole program and evaluation)** ***n*** (%)^**^
Program component?^*^	Implementation support strategies physical activity practices evaluation	N/A 3 (100) N/A	16 (100) N/A N/A	N/A N/A 1 (100)	16 (80) 3 (15) 1 (5)
Proposed impact on the project?	Positive Negative Null Not applicable	2 (67) 1 (33) 0 (0) 0 (0)	13 (81) 3 (19) 0 (0) 0 (0)	0 (0) 0 (0) 0 (0) 1 (100)	15 (79) 4 (21) 0 (0) 1
Relationship to fidelity/core elements?	Fidelity consistent Fidelity inconsistent Not applicable	3 (100) 0 (0) 0 (0)	16 (100) 0 (0) 0 (0)	N/A N/A 1 (100)	19 (100) 0 (0) 1
Were modifications systematic or unsystematic?	Systematic Unsystematic	0 (0) 3 (100)	2 (13) 14 (88)	0 (0) 1 (100)	2 (10) 18 (90)
Were modifications proactive or reactive?	Proactive Reactive	3 (100) 0 (0)	9 (56) 7 (44)	0 (0) 1 (100)	12 (60) 8 (40)
Who participated in the decision to modify?^*^	Program Manager Individual practitioners Treatment/Intervention Team Administrator Recipients None	3 (100) 2 (67) 1 (33) 0 (0) 0 (0) 0 (0)	13 (81) 8 (50) 2 (13) 2 (13) 1 (6) 1 (6)	1 (100) 0 (0) 0 (0) 0 (0) 0 (0) 0 (0)	17 (85) 10 (50) 3 (15) 2 (10) 1 (5) 1 (5)
What was the goal?^*^	Improve fit with recipients Increase satisfaction Improve effectiveness/outcomes Increase reach or engagement Increase retention Improve feasibility None Not applicable	2 (67) 0 (0) 0 (0) 0 (0) 0 (0) 1 (33) 0 (0) 0 (0)	5 (31) 6 (38) 5 (31) 4 (25) 4 (25) 0 (0) 1 (6) 0 (0)	0 (0) 0 (0) 0 (0) 0 (0) 0 (0) 0 (0) 0 (0) 1 (100)	7 (37) 6 (32) 5 (26) 4 (21) 4 (21) 1 (5) 1 (5) 1
What is modified?	Content Training and evaluation Contextual Implementation and scale-up activities	2 (67) 1 (33) 0 (0) 0 (0)	12 (75) 4 (25) 0 (0) 0 (0)	0 (0) 1 (100) 0 (0) 0 (0)	14 (70) 6 (30) 0 (0) 0 (0)
Context modifications are made to what?	Not applicable	3 (100)	16 (100)	1 (100)	20
At what level of delivery were modifications made?	Target intervention group (all schools) Cohort (group of schools sharing a characteristic) Clinic unit/level (individual schools)	3 (100) 0 (0) 0 (0)	11 (69) 3 (19) 2 (13)	1 (100) 0 (0) 0 (0)	15 (75) 3 (15) 2 (10)
What is the nature of the content modification?^*^	Adding elements Tailoring/tweaking/refining Removing/skipping elements Lengthening/extending elements Substituting Reordering of intervention modules or segments Loosening structure Not applicable	0 (0) 1 (33) 1 (33) 0 (0) 0 (0) 0 (0) 0 (0) 1 (33)	5 (31) 2 (13) 1 (6) 1 (6) 1 (6) 1 (6) 1 (6) 4 (25)	0 (0) 0 (0) 0 (0) 0 (0) 0 (0) 0 (0) 0 (0) 1 (100)	5 (36) 3 (21) 2 (14) 1 (7) 1 (7) 1 (7) 1 (7) 6
Reasons – socio-political (i.e., broad context)^*^	None (no reason) Societal/cultural norms Historical context Not applicable	3 (100) 0 (0) 0 (0) 0 (0)	14 (88) 1 (6) 1 (6) 0 (0)	0 (0) 0 (0) 0 (0) 1 (100)	17 (89) 1 (5) 1 (5) 1
Reasons – organization/setting (i.e., PA4E1 implementation team)^*^	Available resources (funds, staff, tech, space) None (no reason) Social context Not applicable	1 (33) 2 (67) 0 (0) 0 (0)	13 (81) 3 (19) 1 (6) 0 (0)	0 (0) 0 (0) 0 (0) 1 (100)	14 (74) 5 (26) 1 (5) 1
Reasons – provider (i.e., Local health district)^*^	None (no reason) Not applicable	3 (100) 0 (0)	16 (100) 0 (0)	0 (0) 1 (100)	19 (100) 1
Reasons – recipient (i.e., Schools and in-school champions)^*^	None (no reason) Motivation and readiness Physical capacity Access to resources Cultural or religious norms Not applicable	1 (33) 2 (67) 0 (0) 0 (0) 0 (0) 0 (0)	8 (50) 5 (31) 3 (19) 2 (13) 1 (6) 0 (0)	0 (0) 0 (0) 0 (0) 0 (0) 0 (0) 1 (100)	9 (47) 7 (37) 3 (16) 2 (11) 1 (5) 1

*Percentages are rounded, so may not sum to 100%*.

*Additional File 4 outlines each individual modification in detail and includes the full set of codes as outlined in the methods*.

* *Percentages may not sum to 100%, and n values may not add up to the number of modifications (N = 20) because multiple codes within this category may be applied to each modification*.

** *“Not applicable (N/A)” category not included in denominator to calculate percentages*.

### Types of Modifications

The vast majority of modifications were “unsystematic” (*n* = 18), because most modifications did not make deliberate use of theory to make the modification. Two modifications made to support sustainability used theory and a specific process, and therefore were deemed systematic. Most modifications (*n* = 12) were made “proactively”, however eight modifications were made in response to an unknown event or circumstance and were therefore coded as “reactive”. Most modifications involved the “program manager” (*n* = 17). “Individual practitioners” participated in half of all decisions (*n* = 10). Other groups and individuals participated in the decision making process less frequently, including the whole PA4E1 Implementation Team “treatment/intervention team” (*n* = 3), local health districts “administrator” (*n* = 2) and in-School Champion “recipients” (*n* = 1).

The goals and types of modifications varied. The most common goals were to “improve fit with recipients” (*n* = 7), “increase satisfaction” (*n* = 6), “improve effectiveness/outcomes” (*n* = 5), “increase reach or engagement” (*n* = 4) and to “increase retention” (*n* = 4). All modifications were to either “content” (*n* = 14) or “training and evaluation” (*n* = 6). The nature of content modifications varied, including “adding elements” (*n* = 5), “tailoring/tweaking/refining” (*n* = 3) and “removing/skipping elements” (*n* = 2). Most modifications (*n* = 15) were made across all schools, in all local health districts, and were therefore coded the “target intervention group” level. The remaining modifications occurred at particular local health districts “cohort level” (*n* = 3) or individual schools “clinic/unit level” (*n* = 4).

### Reasons for Modifications

Reasons for modifications were primarily related to the “available resources” (funds, staff, tech, space) (*n* = 14) of the PA4E1 Implementation Team, i.e., the “provider”. The second most common reason was schools and in-School Champions (i.e., recipient) “motivation and readiness” (*n* = 8). Further reasons for modification were few, but included “historical context” (*n* = 1) and the “social context” (*n* = 1) surrounding the “PA4E1 Implementation Team”.

## Discussion

### Principal Findings

To the authors' knowledge, this is the first study to use a taxonomy to comprehensively report the number, types and reasons for both *adaptations* made to scale-up a program and also the *modifications* that occurred during the delivery of the scaled-up program. Our findings show that 20 *adaptations* were made to the PA4E1 program for scale-up (26, 27, 29), including eight adaptations to the physical activity practices, six to the implementation support strategies and six to the evaluation methods. Most adaptations were proposed to have a positive impact on the effectiveness of the program (*n* = 12). Additionally, 20 *modifications* were made during the delivery of the scaled-up program, of which 16 were proposed to have a positive impact on the effectiveness of the program. Most modifications were to the implementation support strategies (*n* = 16). Given that the use of adaptation and modifications data is being encouraged to explain the findings of scale-up trials (20), the findings of this study provide valuable data, together with detailed process evaluation data (32) to help to explain the findings of the scale-up trial (18, 32).

“Funding and resource availability” was a common reason adaptations and modifications were made. The occurrence of adaptations as part of a research trial, involving the original trial developers, and with a good understanding of the programs and its mechanism of effect may also explain the frequency with which they were fidelity consistent, and thought to have a beneficial impact. The findings underscore the importance of selecting programs that are congruent with the available resources to deliver them at scale, and in doing so, reduce the need for significant adaptations. The use of scalability assessment tools may assist policy makers and practitioners can assist with this process (43).

Both adaptations and modifications to the scale-up of PA4E1 were primarily made to “improve fit with recipients” (i.e., schools). Most adaptations were coded as “fidelity consistent” and “systematic” (i.e., informed by theory and used a process). Such findings are perhaps unsurprising, given they occurred in the context of a funded trial, involving the original trial developers with a good understanding of the program and its mechanism and who employed a considered process to informing adaptations (30). It is also consistent with previous reviews of public health programs (15). Adaptability is a key component of scalable programs, where optimal adaptations are those which are made to fit different contexts and environments while retaining fidelity consistency (12, 13, 21, 43).

Modifications, in contrast to adaptations, were found to be “unsystematic”, likely reflecting the rapid and reactive contexts in which these changes were made by school staff (in-School Champions) and practitioners delivering the program (Support Officers, Program Managers). Previous research into public health prevention programs has similarly found that modifications were unsystematic, suggesting they may be more likely to detract from intervention core functions, resulting in negative impacts on implementation-effectiveness outcomes (44, 45). However, modifications to the program were all coded as “fidelity consistent”, and so retain the intended core functions of the program (outlined in Additional File 3). School staff may have, as a result of the training undertaken as part of the scale-up strategy, or via their existing tacit knowledge, have a good understanding of how the program may impact on the intended outcome and been mindful of this when undertaking modifications. Further research is warranted to explore and better explain such findings.

Reviews of scaled-up physical activity (8) and obesity programs (16) that characterized the nature of adaptations made for scale-up, concluded that adaptations to the “mode of delivery” of programs were particularly prevalent (8, 14, 16). Similarly, we found 13 content adaptations to scale-up PA4E1 and 14 content modifications during delivery of the scaled-up PA4E1.While changes to the delivery modes or content modifications may be perceived as fidelity consistent or improving the overall impacts of a program, for example, by increasing reach and the number of people who may benefit, they may also reduce the absolute effect size of a program (8, 46, 47). That is, the “voltage drop” phenomena whereby the effect sizes of physical activity programs are reduced at scale, may be acceptable from a population perspective if the scaled-up program is capable of reaching and so benefiting (due to delivery mode adaptations) more people, at lower relative cost. Taking a population-level perspective is therefore important when weighing and assessing the potential impact of adaptations or modifications.

### Strengths and Limitations

It has been recommended that researchers consider potential causal pathways of modifications, considering both the intended and unintended impacts of modifications on outcomes (20). In line with our process evaluation protocol (32), we have comprehensively described the extent, type and reasons for adaptations and modifications to PA4E1. A strength of this study is the use of real-time tracking of modifications during delivery to record deviations from the planned protocols, which is expected during trial delivery but often not documented well. Indeed, we found the method to be feasible and informative within our study. We found coding using the FRAME to initially be quite difficult, despite the existence of a coding manual (48). We would suggest to future researchers to consider annotating the FRAME framework coding for their own context (13, 48). We also emphasize the importance of going beyond published papers to generate a list of adaptations. By also using the knowledge of those involved in both the efficacy and scale-up trials, we were able to code more accurately the reasons for adaptation. However, a limitation of our research is that we were not blinded to the outcomes of the implementation-effectiveness trial outcomes, which may have influenced our interpretation (18). Additionally, although this study drew upon historical knowledge of those involved in delivering both the efficacy and scale-up trials, it is possible that some specific details were forgotten, given that the efficacy trial was completed in 2014 (7 years ago). Future studies reporting adaptations should therefore aim to do so prospectively (13, 18). Finally, we used the FRAME to report adaptations and modifications to the physical activity practices, implementation support strategies and evaluation methods. Subsequent to our data analysis, the FRAME-IS (Implementation Strategy) was released which is designed for implementation support strategies and organized into modules. The use of both the FRAME (13) and FRAME-IS (22) may have improved our coding of adaptations and reduced the frequency of consensus meetings required. Additional support to use FRAME to code adaptations and modifications to evaluation methods may also be useful.

## Conclusions

Adaptations and modifications to public health programs are common. Modifications have the potential to impact the implementation and effectiveness of programs. Our findings underscore the importance of comprehensive reporting of the extent, type and reasons for modifications as part of process evaluations, as this data may be important to the interpretation of trial findings. Making modifications that retain core components but better suit a particular context (program adaptability) is considered to be an important component of successfully scaled-up programs. However, it will be important for future programs to identify project management strategies to mitigate the occurrence of reactive operational modifications that are fidelity inconsistent.

Describing the extent, type and reasons for adaptations and modifications made to public health programs provides valuable process evaluation data to help explain the findings of the program. For example, the data may be used to explain the expected reduction in effect size when they are scaled-up. The comprehensive and transparent description of adaptations and modifications will assist us to generate hypotheses relating to the trial process evaluation and implementation outcome data, which will be explored further.

## Data Availability Statement

The original contributions presented in the study are included in the article/Supplementary Material, further inquiries can be directed to the corresponding author/s.

## Ethics Statement

The studies involving human participants were reviewed and approved by Hunter New England Area Human Research Ethics Committee. Written informed consent from the participants' legal guardian/next of kin was not required to participate in this study in accordance with the national legislation and the institutional requirements.

## Author Contributions

MM: conceived the design of the study, led the data analysis, and led the development of the manuscript. MM, JW, EC, RS, and LW: developed the research questions, these were further refined by MM, EC, and LW. RS, EC, LW, and JW: obtained funding for the research. MM: led the ongoing data collection, with significant contributions from EC, RS, TM, and LD. MM, EC, RS, TM, and LD: were involved in the coding of modifications. All authors contributed to the interpretation of findings, provided critical comment on multiple versions of the manuscript, and approved the final manuscript.

## Funding

This project is funded by the NSW Ministry of Health, Translational Research Grant Scheme. The NSW Ministry of Health has not had any role in the design of the study as outlined in this protocol and will not have a role in data collection, analysis of data, interpretation of data and dissemination of findings. This work was also supported by Cancer Council New South Wales. The project received infrastructure support from the Hunter Medical Research Institute (HMRI). RS is supported by a NHMRC TRIP Fellowship (APP1150661). LW is supported by a NHMRC Career Development Fellowship (APP1128348), Heart Foundation Future Leader Fellowship (101175) and a Hunter New England Clinical Research Fellowship.

## Conflict of Interest

The authors declare that the research was conducted in the absence of any commercial or financial relationships that could be construed as a potential conflict of interest.

## Publisher's Note

All claims expressed in this article are solely those of the authors and do not necessarily represent those of their affiliated organizations, or those of the publisher, the editors and the reviewers. Any product that may be evaluated in this article, or claim that may be made by its manufacturer, is not guaranteed or endorsed by the publisher.

## Abbreviations

AHMRC: Aboriginal Health and Medical Research Council
FRAME: Framework for Reporting Adaptations and Modifications-Enhanced
FRAME-IS: Framework for Reporting Adaptations and Modifications-Enhanced - Implementation HMRI: Hunter Medical Research Institute
HNELHD: Hunter New England Local Health District
MADI: Model for Adaptation Design and Impact
NESA: New South Wales Education Standards Authority
NSW: New South Wales
PA: Physical Activity
PA4E1: Physical Activity 4 Everyone
PE: Physical Education
RT4T: Resistance Training 4 Teens
SAAFE, Supportive, Active, Autonomous: Fair and Enjoyable
SERAP: State Education Research Applications Process.
