# Supplementary material for: Extent, Type and Reasons for Adaptation and Modification When Scaling-Up an Effective Physical Activity Program: Physical Activity 4 Everyone (PA4E1)
Source: Front Health Serv. 2021 Nov 17;1:719194. doi: 10.3389/frhs.2021.719194 (PMC10062321; doi:10.3389/frhs.2021.719194)
Supplement: Supplementary file 3 [file Table_3.docx]

**Additional File 3:** Adaptations for ‘scale-up’ of PA4E1 from efficacy to scale-up trial.

| **#** | **Description of the modification** (scale-up from efficacy trial to scale-up trial) *(free text description)* | **Program Component(s)** *(Implementation Support Strategy^, Physical Activity Practice^#^ or Evaluation)* | **Were adaptations systematic and proactive?*** *(FRAME framework codes)* | **Who participated in the decision to modify?** *(FRAME framework codes)* | **What was the goal?** *(FRAME framework codes)* | **What is modified?** *(FRAME framework codes)* | **Context modifications are made to…?***(FRAME framework codes)* | **At what level of delivery?** *(FRAME framework codes)* | **What is the nature of the content modification?** *(FRAME framework codes)* | **Relationship to fidelity/core elements?** *(FRAME framework codes)* | **Reasons – socio-political** (broad context) *(FRAME framework codes)* | **Reasons – organisation/setting** (PA4E1 Implementation Team) *(FRAME framework codes)* | **Reasons – provider** (Local Health Districts and Support Officers) *(FRAME framework codes)* | **Reasons – recipient** (schools and in-School Champion) *(FRAME framework codes)* | **Proposed impact on the project?** (positive, negative or null) |
| --- | --- | --- | --- | --- | --- | --- | --- | --- | --- | --- | --- | --- | --- | --- | --- |
| 1 | **In the efficacy trial**, four of the seven physical activity practices (the evidence based program) were delivered in the first program year, and all seven in the second year. Practice one, two, five and seven were delivered first (1).Therefore enhanced school sport (three), school physical activity policy (four) and linking with the community (six) were not implemented by schools in the first 12 months, but were introduced in the second 12 months of the program.  **In the scale-up trial**, all seven physical activity practices were a part of the program from the start, however, these were delivered incrementally as ‘milestones’ whereby schools started small (e.g. term 1: recess and lunchtime activities offered once a week) and increased this each school term (e.g. term 4: recess and lunchtime activities offered thrice a week). Therefore practice implementation builds over two school years (8 school terms) and is designed to be ongoing (2). | Practices 1-7 | Unsystematic  Proactive | Program manager  (PA4E1 manager)  Treatment/ intervention team  (PA4E1 Implementation Team, inclusive of program manager, project staff, expert advisory group) | Increase reach or engagement  Improve fit with recipients | Contextual | Format | Target intervention group | N/A | Fidelity consistent | None | None | None | Physical capacity  Motivation and readiness | Positive |
| 2 | Practice 1 in the efficacy trial was called “teaching strategies to maximize students’ physical activity in health and PE lessons”. This was subsequently renamed to “Quality Physical Education (PE) lessons” in the scale-up trial.  **In the efficacy trial**, this included teachers leading two pedometer based lessons per teacher each term. Teachers received training and resources to maximize activity in PE including pedometers (lessons to allow monitoring of activity) and workshop learnings including principles for PE teaching (EAASE- Efficient, Active, Autonomous, Success, Enjoyable)  **In the scale-up trial**, the practice included three components. Firstly, that the PE Department used documented principles or guidelines for teachers to maximize PE quality, active learning time and student engagement in PE lessons (program schools used the SAAFE principles – Supportive, Active, Autonomous, Fair and Enjoyable) (3). Secondly, that each PE teacher participated in peer observation of a practical PE lesson, at least once per year. Thirdly, it was desirable (not mandatory to meet the criteria for the practice) that peer observation was against the departments quality PE principles. The focus was on the principles overall rather than primarily about activity levels. | Practice 1 | Systematic  Proactive | Program manager  (PA4E1 manager)  Treatment/ intervention team  (PA4E1 Implementation Team, inclusive of program manager, project staff, expert advisory group) | Increase effectiveness/ outcomes | Content | N/A | Target intervention group | Substituting elements | Fidelity inconsistent  (focus from intensity shifted towards SAAFE PE lessons) | Funding or resource allocation/ availability (the SAAFE resource became available) | None | None | None | Positive |
| 3 | Practice 2 in the efficacy trial was called “Development and monitoring of student physical activity plans within PE lessons”. This was subsequently renamed to “Student physical activity plans” in the scale-up trial.  **In the efficacy trial,** this included Grade seven students in year one and Grade eight students in year two developing individual physical activity plans that set goals and actions and recorded progress against timelines, fitness assessments, and provision of rewards. Plans were to be reviewed and modified each school term.  **In the scale-up trial**, all students in Grade seven in year one, and students in Grade seven and eight in year two, developed a personal physical activity plan which included personal goals to improve or maintain fitness, actions on achieving these goals, and monitoring of progress. Goals were to be reviewed once per school year. | Practice 2 | Systematic  Proactive | Program manager  (PA4E1 manager)  Treatment/ intervention team  (PA4E1 Implementation Team, inclusive of program manager, project staff, expert advisory group) | Improve fit with recipients | Content | N/A | Target intervention group | Shortening/ condensing | Fidelity consistent | None | None | None | Physical capacity | Positive |
| 4 | Practice 3 in both trials was called “Enhanced school sport program”.  **In the efficacy trial**, the practice was for all Grade eight students to participate in a 10-week program during school sport. The program was based on the effective ‘Program X’ (4) and included lessons and fitness activities focused on lifelong physical activity skills and knowledge.  **In the scale-up trial**, the enhanced school sport program was also a 10-12 week structured program during school sport. The program was new, ‘Resistance Training for Teens’ (RT4T) (5), and was offered as accredited training by the NSW Department of Education. It was to be delivered to all students in at least Grade 7. The cost of participating was covered for the in-School Champion, and other teachers who were to deliver the program. | Practice 3 | Systematic  Proactive | Program manager  (PA4E1 manager)  Treatment/ intervention team  (PA4E1 Implementation Team, inclusive of program manager, project staff, expert advisory group) | Improve fit with recipients | Content | N/A | Target intervention group | Substituting | Fidelity consistent | Funding or resource availability/ allocation.  (the resource RT4T became available) | None | None | None | Positive |
| 5 | Practice 4 in the efficacy trial was called “development/modification of school policies”. This was subsequently renamed to “School Physical Activity Policy or Procedure” in the scale-up trial. In the scale-up trial, this practice became practice number 5.  **In the efficacy trial**, schools were to develop or modify existing school policies that aimed to enhance student physical activity through collaboration between the head PE teacher, in-school consultant (support strategy 1), and school executive.  **In the scale-up trial**, schools were asked to develop a school physical activity policy that included provision of at least 150 minutes of moderate to vigorous intensity physical activity during school time for all students in Grade 7-10 as well as a reference to the other physical activity practices of the program. | Practice 4 | Systematic  Proactive | Program manager  (PA4E1 manager)  Treatment/ intervention team  (PA4E1 Implementation Team, inclusive of program manager, project staff, expert advisory group) | Increase satisfaction  (i.e. give clearer guidance to schools on developing a policy) | Content | N/A | Target intervention group | Substituting | Fidelity consistent | Existing policies | None | None | None | Positive |
| 6 | Practice 5 in the efficacy trial was called “physical activity programs during school breaks”. In the scale-up trial, this was subsequently renamed to “recess/ lunchtime physical activity”.  **In the efficacy trial**, schools were encouraged to offer teacher supervised physical activity at recess and lunchtime on at least 2 days per week.  **In the scale-up trial**, schools were asked to offer all students in Grades 7-10 recess and/or lunchtime activities on at least 3 days per week. As well, schools were asked to provide access to physical activity equipment to students at least 3 days per week. It was desirable that at least one organized recess or lunchtime activity specifically targeted girls. It was also desirable that sessions be promoted to students at least once per term. | Practice 5 | Systematic  Proactive | Program manager  (PA4E1 manager)  Treatment/ intervention team  (PA4E1 Implementation Team, inclusive of program manager, project staff, expert advisory group) | Improve effectiveness/ outcomes | Content | N/A | Target intervention group | Lengthening/ extending  Adding elements | Fidelity consistent | None | None | None | Access to resources  (in-School Champion funded) | Positive |
| 7 | Practice 6 in the efficacy trial was called “promotion of community physical activity providers (community links)”. In the scale-up trial, this was subsequently renamed to “links with community physical activity providers”.  **In the efficacy trial**, schools hosted a physical activity exhibition that promoted local physical activity providers to students in Grade 8.  **In the scale-up trial**, school was asked to form three links with community physical activity providers that go beyond promotion of the provider (e.g. in newsletter) to involve an agreement, connection or partnership (e.g. presentation at the schools, sessions outside of school hours run at the school). The links were to support outside of school time activity and were communicated to students and families at least once per term. It was desirable that at least one of the community links made were to promote free or low cost options in the community. Schools were asked to use multiple modes to promote (e.g. newsletter, parent app) | Practice 6 | Systematic  Proactive | Program manager  (PA4E1 manager)  Treatment/ intervention team  (PA4E1 Implementation Team, inclusive of program manager, project staff, expert advisory group) | Improve fit with recipients | Content | N/A | Target intervention group | Substituting | Fidelity consistent | None | None | None | Access to resources  (in-School Champion funded) | Positive |
| 8 | Practice 7 in the efficacy trial was called “parent engagement”. In the scale-up trial, this was subsequently renamed to “communicating physical activity messages to all parents”.  **In the efficacy trial**, schools were to send information to parents via newsletters and the school website promoting physical activity and local providers, once per term.  **In the scale-up trial**, schools were to send physical activity messages to parent that were designed to increase parent knowledge, attitudes and support for physical activity, at least once per term, to parents of students in Grade 7-10. Schools were asked to use multiple modes to communicate the messages (e.g. newsletter, parent app) | Practice 7 | Systematic  Proactive | Program manager  (PA4E1 manager)  Treatment/ intervention team  (PA4E1 Implementation Team, inclusive of program manager, project staff, expert advisory group) | Increase effectiveness/ outcomes | Content | N/A | Target intervention group | Lengthening/ extending | Fidelity consistent | Societal/ cultural norms | None | None | None | Positive |
| 9 | Implementation Strategy 1 in the efficacy trial was called “in-school physical activity consultant”. In the scale-up trial, this was subsequently split into two implementation support strategies, named “Embedded school staff: in-School Champion” and “External Implementation Support”. These two strategies were also renumbered to implementation strategies 2 and 3 respectively.  **In the efficacy trial**, a trained PE teacher was placed within each school (in-school physical activity consultant) for 1 day per week over the program period to support program implementation.  **In the scale-up trial**, instead of an external consultant visiting the school, an existing physical education (PE) teacher at the school was identified by the school (usually a decision for the Head PE teacher and school executive) to lead the role of in-School Champion. The in-School Champion was supported by a Health Promotion Support Officer employed by their respective local health district. The in-School Champion was an existing school PE teacher who allocated the role of in-School Champion to support implementation for full 24 months. In the event an in-School Champion left the school, a new in-School Champion was identified by the school to lead the program within their school. The role of in-School Champion was funded by the NSW Department of Health, half day per week (equivalent to $400AUD a fortnight).  **Summary of adaptations:**   - External physical activity consultant replaced by an in-School Champion who was supported by a Health Promotion Support Officer - In -School Champions were funded $400 a fortnight - Support Officer and in-School Champion maintained contact through face-to-face meetings, email and phone according to the schedule documented within the support strategy. - Support Officer was co-located in the same local health district with in-School Champions | Implementation strategy 1 | Systematic  Proactive | Program manager  (PA4E1 manager)  Treatment/ intervention team  (PA4E1 Implementation Team, inclusive of program manager, project staff, expert advisory group) | Improve feasibility  Improve fit with recipients | Content  Implementation and scale-up activities | N/A | Target intervention group | Substituting | Fidelity inconsistent | None | Available resources (funds, staffing, technology, space) | None | Cultural or religious norms  Motivation and readiness | Positive |
| 10 | Implementation Strategy 2 in the efficacy trial was called “establishing leadership and support”. In the scale-up trial, this was subsequently renamed to “Executive and leadership support”. This strategy was also renumbered to implementation strategy 1.  **In the efficacy trial**, a school committee was established, including school executive, Head PE teacher, community representative, student representative and parent representative, or responsibility was added to an existing committee, to lead and oversee the program. Schools executives were asked to sign a partnership agreement. Committee to meet once per term.  **In the scale-up trial**, schools were asked to get the school executive to sign a partnership agreement. Additionally, a school committee was established, or responsibility was added to an existing committee, to lead and oversee the program, meeting once per term and inclusive of the in-School Champion and school executive.  **Summary of adaptations:**   - Less total committee members in the scale-up trial (i.e. no requirement for student, parent, Head PE teacher and community representative) and include both the in-School Champion and school executive. | Implementation strategy 2 | Systematic  Proactive | Program manager  (PA4E1 manager)  Treatment/ intervention team  (PA4E1 Implementation Team, inclusive of program manager, project staff, expert advisory group) | Improve fit with recipients | Content  Implementation and scale-up activities | N/A | Target intervention group | Shortening/ condensing | Fidelity consistent | None | Available resources (funds, staffing, technology, space) | None | Cultural or religious norms | Positive |
| 11 | Implementation Strategy 3 in the efficacy trial was called “teacher training”. In the scale-up trial, this was subsequently renamed to “teacher professional learning”. This strategy was also renumbered to implementation strategy 4.  **In the efficacy trial**, PE teachers were offered three two-hour face-to-face practice learning workshops (all schools together) focused on delivery of lessons to increase students’ moderate-to-vigorous physical activity (MVPA). All PE teachers and teachers involved in the delivery of the enhanced school sports program were invited to face-to-face training (at least once PE teacher per school to attend- all invited).  **In the scale-up trial**, not all training was delivered face-to-face, and not all PE Teachers were invited to all training. A website was created to deliver online training to PE teachers, whereas in-School Champions also received some face-to-face training. Aspects of the training were also officially accredited with NSW Education Standards Authority (NESA), the accrediting body for teachers in NSW, providing teachers with points towards their five-yearly requirement. Training was split into four components:   - In-School Champion training −3 x 1 day of face-to-face training were hosted by PA4E1 implementation team, in Term 1, 5 and 9. Meals and transport costs were covered by the PA4E1 program (NSW Department of Health) for all three training sessions. Accommodation was covered for the first two workshops, however costs for accommodation were not covered for the final workshop. - School PA policy training – in-School Champion offered existing online training (6 hours) run by the NSW Department of Education School Sport Unit (Government schools only, n = 19). - Enhanced school sport training – in-School Champions and other teachers involved in delivering the program could attend an existing 1 day face-to-face Resistance Training for Teens workshop offered by the NSW Department of Education (School Sport Unit), or equivalent training run by PA4E1 implementation team (not accredited). Course costs to be paid by project for in-School Champion, but not for other teachers. - Quality PE training for all PE teachers (including in-School Champions) - 6 × 10-min online training videos followed by knowledge check short quizzes focused on the SAAFE principles were delivered via a password protected program website.   **Summary of adaptations:**   - PE Teacher training via website rather than face-to-face - NESA accreditation attached to online training - Specific training for writing a physical activity policy for in-School Champions - 3 days training for in-School Champion | Implementation strategy 3 | Systematic  Proactive | Program manager  (PA4E1 manager)  Treatment/ intervention team  (PA4E1 Implementation Team, inclusive of program manager, project staff, expert advisory group) | Improve feasibility  Improve fit with recipients  Increase effectiveness/ outcomes  Reduce cost | Content  Implementation and scale-up activities | N/A | Target intervention group | Substituting  Adding elements | Fidelity consistent | Funding or resource allocation/ availability | Available resources (funds, staffing, technology, space) | None | Cultural or religious norms | Positive |
| 12 | Implementation Strategy 4 in the efficacy trial was called “Resources”. In the scale-up trial, this was also named “Resources”. This strategy was renumbered to implementation strategy 5.  **In the efficacy trial**, schools were provided with AUD$11,874 worth of resources. This included a paper-based manual outlining all physical activity program strategies and associated materials; approximately AUD$6000 of physical activity equipment (e.g., pedometers, resistance devices, games consoles, equipment boxes); and promotional materials for teachers (e.g., shirts/lanyards) and students (e.g., balls, water bottles).  **In the scale-up trial**, fewer physical resources were distributed to schools. Documents were hosted on the program website, rather than distributed as printed copies. Schools received gym sticks to support the enhanced school sport program and a $100 voucher to purchase physical activity equipment. Schools also received printed posters outlining the SAAFE principles, to be displayed in the PE department. Promotional materials were issued to both program and control schools (reusable coffee cups, small balls) as reimbursement for completing evaluation measures, and thus were not considered as part of this support strategy.  **Summary of adaptations:**   - Paper-based resources were replaced by a website housing documentation and resources, except for printed posters outlining the SAAFE principles (for Practice 1) - Less total equipment provided, 5 gymsticks and an equipment voucher was provided to schools rather than providing all the $6000 equipment - Promotional materials were not issued as part of the support strategy, but instead for completion of evaluation measures | Implementation strategy 4 | Systematic  Proactive | Program manager  (PA4E1 manager)  Treatment/ intervention team  (PA4E1 Implementation Team, inclusive of program manager, project staff, expert advisory group) | Improve feasibility  Reduce cost | Content  Implementation and scale-up activities | N/A | Target intervention group | Shortening/ condensing | Fidelity consistent | None | Available resources (funds, staffing, technology, space) | None | None | Negative |
| 13 | Implementation Strategy 5 in the efficacy trial was called “Prompts”. In the scale-up trial, this was subsequently renamed to “Provision of prompts and reminders”. This strategy was also renumbered to implementation strategy 6.  **In the efficacy trial**, the in-school consultant provided prompts to teaching staff to implement the program strategies via e-mail, electronic calendar reminders, and in face-to-face meetings.  **In the scale-up trial**, prompts were delivered by Support Officers to in-School Champions via email, phone and face-to-face to encourage implementation. Prompts were also issued via email automatically via the website each term to in-School Champions and PE teachers to prompt teacher completion of professional learning. In-School Champions were additionally prompted by email to complete termly performance monitoring surveys against the physical activity practices, and received automated feedback surveys from this survey.  **Summary of adaptations:**   - Support Officers reminded in-School Champions to implement the program, rather than the in-school consultant - Automated prompts to in-School Champions and PE teachers delivered via the program website | Implementation strategy 5 | Systematic  Proactive | Program manager  (PA4E1 manager)  Treatment/ intervention team  (PA4E1 Implementation Team, inclusive of program manager, project staff, expert advisory group) | Improve feasibility  Improve fit with recipients | Content  Implementation and scale-up activities | N/A | Target intervention group | Substituting | Fidelity consistent | None | Available resources (funds, staffing, technology, space) | None | Cultural or religious norms | Positive |
| 14 | Implementation Strategy 6 in the efficacy trial was called “intervention implementation performance feedback”. In the scale-up trial, this was subsequently renamed to “implementation performance monitoring and feedback”. This strategy was also renumbered to implementation strategy 7.  **In the efficacy trial**, records kept by the in-school consultant were the basis of quarterly program implementation feedback reports (on how was the school progressing with the practices) provided via email and hard copy to Principals and Head of PE.  I**n the scale-up trial**, automated feedback were generated following in-School Champion completion of termly performance monitoring surveys via the website (whether schools were meeting milestones for each practice). Feedback was issued as a PDF report via email to in-School Champions and school Principals.  **Summary of adaptations:**   - Feedback was automated via the program website, and was directly against the physical activity practice milestones (as practice implementation builds over two school years and is designed to be ongoing) - Feedback was automatically sent to (website registered) in-School Champions and Principals - No direct observations were undertaken by the Support Officer. | Implementation strategy 6 | Systematic  Proactive | Program manager  (PA4E1 manager)  Treatment/ intervention team  (PA4E1 Implementation Team, inclusive of program manager, project staff, expert advisory group) | Improve feasibility  Reduce cost | Content  Implementation and scale up activities | N/A | Target intervention group | Substituting | Fidelity consistent | None | Available resources (funds, staffing, technology, space) | None | None | Null |
| 15 | Both trials used a cluster-randomized controlled trial design.  **The efficacy trial** recruited 10 schools.    **The scale-up trial** planned to recruit up to 78 schools -recruited 49 schools. A nested sub-study involving 30 schools evaluated student level outcomes. The nested sub-study did not include baseline measures (post-test comparisons only) | Evaluation | Systematic  Proactive | Program manager  (PA4E1 manager)  Treatment/ intervention team  (PA4E1 Implementation Team, inclusive of program manager, project staff, expert advisory group) | N/A | Training and Evaluation  Implementation and scale-up activities | N/A | Target intervention group | N/A | N/A | N/A | N/A | N/A | N/A | N/A |
| 16 | **The efficacy trial** primary outcome was daily mean minutes of student moderate-to-vigorous physical activity (MVPA) assessed through accelerometry (hip-worn).  **The scale-up trial** primary outcome is proportion of schools adopting at least four of the seven practices, assessed via Head PE teacher computer-assisted telephone interview (CATI). | Evaluation | Systematic  Proactive | Program manager  (PA4E1 manager)  Treatment/ intervention team  (PA4E1 Implementation Team, inclusive of program manager, project staff, expert advisory group) | N/A | Training and Evaluation  Implementation and scale-up activities | N/A | Target intervention group | N/A | N/A | N/A | N/A | N/A | N/A | N/A |
| 17 | **The efficacy trial** secondary outcomes were student anthropometry measures.  **The scale-up trial** secondary outcomes included student daily mean minutes of MVPA assessed through accelerometry (wrist-worn) and anthropometry in a sub-sample of 30 schools. Other secondary outcomes were mean number of school practices and percentage of schools meeting each practice. | Evaluation | Systematic  Proactive | Program manager  (PA4E1 manager)  Treatment/ intervention team  (PA4E1 Implementation Team, inclusive of program manager, project staff, expert advisory group) | N/A | Training and Evaluation  Implementation and scale-up activities | N/A | Target intervention group | N/A | N/A | N/A | N/A | N/A | N/A | N/A |
| 18 | **The efficacy trial** process evaluation focused on practice uptake assessed through physical activity consultant records in program schools, as well as some focus on fidelity and reach of the program. Unpublished focus groups with school stakeholders were also held.  **The scale-up** **trial** process evaluation was more detailed in design, and a protocol describing it’s design has been published (6). This included a broader focus on modifications, fidelity, reach of the implementation support strategies, and the acceptability, appropriateness and feasibility of the support and program overall. | Evaluation | Systematic  Proactive | Program manager  (PA4E1 manager)  Treatment/ intervention team  (PA4E1 Implementation Team, inclusive of program manager, project staff, expert advisory group) | N/A | Training and Evaluation  Implementation and scale-up activities | N/A | Target intervention group | N/A | N/A | N/A | N/A | N/A | N/A | N/A |
| 19 | **The efficacy trial** was delivered in three local health districts across NSW (Hunter New England, Central Coast and Mid North Coast).  **The scale-up trial** was additionally delivered in South Western Sydney. | Evaluation | Systematic  Proactive | Program manager  (PA4E1 manager)  Treatment/ intervention team  (PA4E1 Implementation Team, inclusive of program manager, project staff, expert advisory group) | N/A | Training and Evaluation  Implementation and scale-up activities | N/A | Target intervention group | N/A | N/A | N/A | N/A | N/A | N/A | A |
| 20 | **The efficacy trial** cost effectiveness evaluation focused on cost and incremental cost effectiveness ratios for the following: minutes of MVPA per day gained, metabolic equivalent of task (MET) hours gained per person/day; Body Mass Index (BMI) unit avoided; and 10 % reduction in BMI z-score.  **The scale-up trial instead** planned additional cost analyses, including a costs, cost consequence analysis, cost effectiveness analysis and a budget impact analysis. This will include cost effectiveness analyses focused on the practice uptake (primary trial outcome) – as well as on student level outcomes. | Evaluation | Systematic  Proactive | Program manager  (PA4E1 manager)  Treatment/ intervention team  (PA4E1 Implementation Team, inclusive of program manager, project staff, expert advisory group) | N/A | Training and Evaluation  Implementation and scale-up activities | N/A | Target intervention group | N/A | N/A | N/A | N/A | N/A | N/A | N/A |

**Footnotes:**

Modifications were coded to the Stirman et al Framework, ‘FRAME’ (7). Two meetings were held in February 2021 between the implementation team (Support Officers) and the researchers to reach consensus on the final codes.

*Based on the MADI framework (8), we revised the FRAME framework (7) wording to remove concept of “planned” and replace with concept of “systematic.” This emphasizes the importance of how the adaptation was made (i.e., was it done using a systematic process), in addition to whether the adaptation was proactive (made due to an anticipated obstacle) or reactive (due to unanticipated challenges).

^Implementation Support Strategies 1.1-7.3 are outlined in Sutherland et al 2020 (9).

^#^ Physical Activity Practices (1-7) are outlined in Sutherland et al 2020 (9).

Abbreviations:

N/A = Not applicable;

PA4E1: Physical Activity 4 Everyone

SAAFE: Supportive, Active, Autonomous, Fair and Enjoyable

EAASE: Efficient, Active, Autonomous, Success, Enjoyable

NSW: New South Wales

PE: Physical Education

FRAME: Framework for Reporting Adaptations and Modifications-Enhanced

MADI: Model for Adaptation Design and Impact

MVPA: moderate-to-vigorous physical activity

MET: metabolic equivalent of task

BMI: Body Mass Index

RT4T: Resistance Training 4 Teens

CATI: computer-assisted telephone interview

NESA: New South Wales Education Standards Authority

**References**

1. Sutherland R, Campbell L, Lubans D, Morgan P, Okely AD, Nathan N, et al. ‘Physical Activity 4 Everyone’ school-based intervention to prevent decline in adolescent physical activity levels: 12 month (mid-intervention) report on a cluster randomised trial. British Journal of Sports Medicine. 2015.

2. Sutherland R, Campbell E, Nathan N, Wolfenden L, Lubans DR, Morgan PJ, et al. A cluster randomised trial of an intervention to increase the implementation of physical activity practices in secondary schools: study protocol for scaling up the Physical Activity 4 Everyone (PA4E1) program. BMC Public Health. 2019;19(1):883.

3. Lubans DR, Lonsdale C, Cohen K, Eather N, Beauchamp MR, Morgan PJ, et al. Framework for the design and delivery of organized physical activity sessions for children and adolescents: rationale and description of the ‘SAAFE’ teaching principles. International Journal of Behavioral Nutrition and Physical Activity. 2017;14(1):24.

4. Lubans DR, Morgan PJ, Callister R, Collins CE, Plotnikoff RC. Exploring the mechanisms of physical activity and dietary behavior change in the program x intervention for adolescents. The Journal of adolescent health : official publication of the Society for Adolescent Medicine. 2010;47(1):83-91.

5. Kennedy SG, Smith JJ, Morgan PJ, Peralta LR, Hilland TA, Eather N, et al. Implementing Resistance Training in Secondary Schools: A Cluster Randomized Controlled Trial. Medicine and science in sports and exercise. 2018;50(1):62-72.

6. McLaughlin M, Duff J, Sutherland R, Campbell E, Wolfenden L, Wiggers J. Protocol for a mixed methods process evaluation of a hybrid implementation-effectiveness trial of a scaled-up whole-school physical activity program for adolescents: Physical Activity 4 Everyone (PA4E1). Trials. 2020;21(1):268.

7. Wiltsey Stirman S, Baumann AA, Miller CJ. The FRAME: an expanded framework for reporting adaptations and modifications to evidence-based interventions. Implementation Science. 2019;14(1):58.

8. Kirk MA, Moore JE, Wiltsey Stirman S, Birken SA. Towards a comprehensive model for understanding adaptations’ impact: the model for adaptation design and impact (MADI). Implementation Science. 2020;15(1):56.

9. Sutherland R, Campbell E, McLaughlin M, Nathan N, Wolfenden L, Lubans DR, et al. Scale-up of the Physical Activity 4 Everyone (PA4E1) intervention in secondary schools: 12-month implementation outcomes from a cluster randomized controlled trial. International Journal of Behavioral Nutrition and Physical Activity. 2020;17(1):100.
