# Supplementary material for: Extent, Type and Reasons for Adaptation and Modification When Scaling-Up an Effective Physical Activity Program: Physical Activity 4 Everyone (PA4E1)
Source: Front Health Serv. 2021 Nov 17;1:719194. doi: 10.3389/frhs.2021.719194 (PMC10062321; doi:10.3389/frhs.2021.719194)
Supplement: Supplementary file 4 [file Table_4.docx]

**Additional File 4:** Modifications made during PA4E1 implementation trial

| **#** | **Description of the modification** | **Reason for the modification** | **Implementation Support Strategy^ or Physical Activity Practice** | **Term(s) modification made/notified to Schools (Terms 1-9)** | **Were adaptations systematic and proactive?*** | **Who participated in the decision to modify?** | **What was the goal?** | **What is modified?** | **Context modifications are made to…?** | **At what level of delivery?** | **What is the nature of the content modification?** | **Relationship to fidelity/core elements?** | **Reasons – socio-political** | **Reasons – organisation/setting (PA4E1 Implementation Team**)** | **Reasons – provider (LHD)** | **Reasons – recipient (Schools)** | **Proposed impact on the project?**  (positive, negative or null) |
| --- | --- | --- | --- | --- | --- | --- | --- | --- | --- | --- | --- | --- | --- | --- | --- | --- | --- |
| 1 | For the entire duration of the program, funding for in-School Champions was increased from AUD$350 a fortnight to AUD$400. | To align with the cost of a relief teacher. The cost of a relief teacher to relieve in-School Champions of teaching duty was determined to be approximately $400. | 2.2 | Term 1 | Unsystematic and proactive | Program manager  Treatment/Intervention Team | Improve fit with recipients | Content | N/A | Target intervention group  (All schools) | Tailoring/tweaking/refining | Fidelity consistent | None | Available resources (funds, staffing, technology, space) | None | Access to resources | Positive – providing schools with the money to cover the cost of a relief teacher |
| 2 | Physical resources were issued as 'incentives' upon completion of training, though all schools ended up receiving the resources as they all completed the necessary training. All schools ended up receiving the resources if they wanted them. | The implementation team always planned on offering resources in this way, but this wasn't written apriori into the protocol. | 5.2, 5.3 | Term 1-5 | Unsystematic and proactive | Program manager | Improve effectiveness/ outcomes  Increase reach or engagement | Content | N/A | Target intervention group  (All schools) | Reordering of intervention modules or segments | Fidelity consistent | None | None | None | Motivation and readiness | Positive – it was designed to motivate schools to complete training by incentivising them with training. |
| 3 | Support Officer not co-located within same Local Health District. | Staffing availability in two Local Health Districts. | 3.2 | Term 1 | Unsystematic and reactive | Program manager  Administrator | Increase retention | Content | N/A | Cohort level  (All schools in two Local Health Districts) | Loosening structure | Fidelity consistent | None | Available resources (funds, staffing, technology, space) | None | None | Negative - Potential to reduce the number of face-to-face contacts between Support Officers and in-School Champions. |
| 4 | Support Officer not trained in physical education teaching. | Staffing availability in one local health district. | 3.1 | Term 1 | Unsystematic and reactive | Program manager  Administrator | Increase retention | Content | N/A | Cohort level  (All schools within the Local Health District) | Substituting | Fidelity consistent | None | Available resources (funds, staffing, technology, space) | None | None | Negative - Potential to reduce the rapport and understanding between the Support Officer and in-School Champions. |
| 5 | Prompting emails reminding users to complete professional development were not sent to PE Teacher or in-School Champion users of the website if they registered after the first term of the program. An error by the PA4E1 Implementation Team. | An accidental oversight by the program developers in the coding of automated emails. It could not be fixed without considerable cost. | 6.2 | Term 2 | Unsystematic and reactive | None | None | Content | N/A | Target intervention group  (All schools) | Removing/ skipping elements | Fidelity consistent | None | Available resources (funds, staffing, technology, space) | None | None | Negative – schools with higher staff turnover (i.e. more new PE teachers and in-School Champions) would not receive prompts to complete professional development due to the coding error in the automated email system. |
| 6 | A set of 30 pedometers were made available to schools who wanted them. Not all schools wanted them. We had 3 sets available. | Schools were able to use a set of pedometers, from the PA4E1 program, for a term.  The implementation team had planned on offering resources in this way, but this wasn't written apriori into the protocol. | 5 | Term 4 | Unsystematic and proactive | Program manager  Treatment/intervention team | Improve effectiveness/outcomes  Increase satisfaction | Content | N/A | Target intervention group  (All schools) | Adding elements | Fidelity consistent | None | Available resources (funds, staffing, technology, space) | None | None | Positive - Schools who received the pedometers received more support than schools who didn’t receive the pedometers. |
| 7 | Enhanced school sport training delivered by Support Officers to a single school as Department of Education training dates had expired. In-School Champions and PE Teachers unable to receive accreditation for this ad-hoc training. | To allow schools who missed the training, or teachers new to the schools, to take part in enhanced school sport training when the Department of Education were not running it. | 3 | Term 4 | Unsystematic and reactive | Program manager    Individual practitioners | Improve fit with recipients  Improve effectiveness/outcomes | Training and Evaluation | N/A | Clinic/Unit Level | N/A | Fidelity consistent | None | Available resources (funds, staffing, technology, space) | None | Physical capacity  Motivation and readiness | Positive – to maintain high fidelity to the training for schools unable to attend the main face-to-face training events. |
| 8 | Extra day of face-to-face training held halfway through the program (Term 6). While a second day of face to face training was outlined within the study protocol (Table 2, Sutherland et al 2019 (1)), School Champions were not made aware of this until Term 4 of the program. This was because the program team were unsure about available resources. | To re-motivate and engage in-School Champions. We also used it for evaluation purposes to conduct focus groups. | 4.1 | Term 4 | Unsystematic and proactive | Program manager | Improve effectiveness/outcomes  Increase satisfaction  Increase retention | Training and Evaluation | N/A | Target intervention group  (All schools) | N/A | Fidelity consistent | None | Available resources (funds, staffing, technology, space)  Social context (culture, climate, leadership support) | None | Physical capacity  Motivation and readiness | Positive – In-School Champions enjoyed and found useful the additional opportunity to meet face-to-face. |
| 9 | Facebook Group created by in-School Champions to facilitate resource, discussion and knowledge exchange. | In-School Champions thought that it may be better to use Facebook than the PA4E1 Online discussion forum. | 5.4 | Term 5 | Unsystematic and proactive | Recipients  Individual practitioners | Improve fit with recipients  Increase satisfaction | Content | N/A | Target intervention group  (All schools) | Adding elements | Fidelity consistent | Societal/cultural norms | None | None | Cultural or religious norms | Positive – In-School Champions led the request to create a private Facebook group to facilitate knowledge and resource sharing. |
| 10 | Physical activity plans to be completed by Grade 7 only in the second half of the program, not both Grade 7 and 8 (as originally described in the protocol). | To increase the feasibility of running the physical activity plans activity following feedback from in-School Champions and PE Teachers. | Practice 2 | Term 5 | Unsystematic and proactive | Program manager  Individual practitioners | Improve fit with recipients | Content | N/A | Target intervention group  (All schools) | Removing/ skipping elements | Fidelity consistent | None | None | None | Motivation and readiness | Negative – students in Grade 7 in the first year of the program would not get another chance to continue with their student physical activity plans in Grade 8. |
| 11 | Face-to-face training was repeated for schools unable to attend the centralised training held for all schools. This training was delivered locally to the schools, to reduce travel times for the in-School Champions. | To ensure that all schools had the opportunity to receive the necessary training, even if they couldn’t attend the main training days. | 4.1 | Term 5 | Unsystematic and reactive. | Program manager  Individual practitioners | Improve fit with recipients | Training and Evaluation | N/A | Cohort level | N/A | Fidelity consistent | None | Available resources (funds, staffing, technology, space) | None | None | Positive – to maintain high fidelity to the training for schools unable to make the main face-to-face training events. |
| 12 | Additional physical resources for enhanced school sport training sent to a single school who requested them from their Support Officer. | To accommodate the request of a specific school who needed additional physical resources (activity cards for the enhanced school sport program). | 5 | Term 6 | Unsystematic and reactive | Individual practitioners | Increase satisfaction  Improve fit with recipients | Content | N/A | Clinic/unit level  (one school) | Adding elements | Fidelity consistent | None | None | None | Access to resources | Positive – school request for additional resources to deliver Practice 3 of the program. |
| 13 | Lesson observation forms could be submitted either through the website form or uploaded as a word document (new). | To make it easier for PE teachers and in-School Champions to submit their lesson observations. | Practice 1 | Term 6 | Unsystematic and proactive | Program manager    Individual practitioners | Improve fit with recipients | Content | N/A | Target intervention group  (All schools) | Tailoring/ tweaking/ refining | Fidelity consistent | None | Available resources (funds, staffing, technology, space) | None | None | Positive – making it easier for PE teachers and in-School Champions to upload lesson observations (peer feedback). |
| 14 | All schools were sent incorrect termly survey feedback reports due to an error with the termly survey. A replacement report was sent with the correct information. | Some schools would have received a lower grade initially, but then the implementation team decided it was important to correct this error for schools. | 7.2, 7.3 | Term 7 | Unsystematic and reactive | Program manager    Individual practitioners | Increase satisfaction | Content | N/A | Target intervention group  (All schools) | Tailoring/ tweaking/ refining | Fidelity consistent | None | Available resources (funds, staffing, technology, space) | None | None | Positive – to rectify a mistake made in the feedback given to schools, this solution aimed to reduce the impact of the mistake. |
| 15 | Termly survey definition of meeting practice 6, changed from having a low or no-cost option community link -from mandatory to desirable. | To increase the feasibility of meeting the practice. | Practice 6 | Term 7 | Unsystematic and proactive | Program manager  Treatment/intervention team | Improve feasibility | Training and Evaluation | N/A | Target intervention group  (All schools) | N/A | Fidelity consistent | None | None | None | Motivation and readiness | Positive - More schools will meet the practice as it is easier to achieve. |
| 16 | Extension of the whole program by one school term, extending the program from 8 school terms to 9 school terms. However, schools were not provided additional funds for release of the in-School Champion (Support Strategy 2.2) | To align the program end with the end of the school year, and to give schools the chance to plan sustainability. | 1-7 | Term 7 | Systematic and proactive | Program manager | Increase reach or engagement | Content | N/A | Target intervention group  (All schools) | Lengthening/ extending | Fidelity consistent | None | Available resources (funds, staffing, technology, space) | None | None | Positive – program was lengthened to increase the likelihood of PA4E1 being sustained within schools. |
| 17 | Extra day of face-to-face training held at the end of the program (Term 9). | To re-motivate and engage in-School Champions to set-up sustainability plans. | 4.1 | Term 7 | Unsystematic and proactive | Program manager  Individual practitioners | Improve effectiveness/outcomes  Increase satisfaction  Increase retention | Training and Evaluation | N/A | Target intervention group  (All schools) | N/A | Fidelity consistent | None | Available resources (funds, staffing, technology, space)  Social context | None | Physical capacity  Motivation and readiness | Positive – In-School Champions enjoyed and found useful the additional opportunity to meet face-to-face. |
| 18 | Sustainability reports, similar to termly surveys and feedback reports, were designed to assist schools to plan strategies for sustaining the PA4E1 program in their school beyond the life of the research project. These were issued via email to be completed by in-School Champions in liaison with their school Principal. | To increase sustainability of the program beyond the length of the research project. | 7.2, 7.3 | Term 8 | Systematic and proactive | Program manager  Individual practitioners | Increase reach or engagement | Content | N/A | Target intervention group  (All schools) | Adding elements | Fidelity consistent | None | Available resources (funds, staffing, technology, space) | None | None | Positive – sustainability reports were designed to assist schools to plan strategies for sustaining the PA4E1 program in their school beyond the life of the research project. |
| 19 | All School Principals were offered a face-to-face meeting in Term 8 to explain their schools 24 month sustainability report. | To increase executive support for PA4E1 and to contribute to sustainability of PA4E1 beyond the research program. | 1  and  7.3 | Term 8 | Unsystematic and proactive | Program manager    Individual practitioners | Increase reach or engagement | Content | N/A | Target intervention group  (All schools) | Adding elements | Fidelity consistent | Historical context | Available resources (funds, staffing, technology, space) | None | Motivation and readiness. | Positive – report could be discussed with Principals to obtain executive support for sustainability strategies for the PA4E1 program. |
| 20 | Postponement, then abandonment of 24 month student data collection | Due to COVID-19 pandemic, there was increasing disruption to usual activity and restricted access to schools/no external people. | Evaluation | Term 6 | Unsystematic and reactive | Program manager | None | Training and Evaluation | N/A | Target intervention group  (All schools) | N/A | N/A | N/A | N/A | N/A | N/A | N/A |

Footnotes:

*Based on the MADI framework (5), we revised the FRAME framework (4) wording to remove concept of “planned” and replace with concept of “systematic.” This emphasizes the importance of how the adaptation was made (i.e., was it done using a systematic process), in addition to whether the adaptation was proactive (made due to an anticipated obstacle) or reactive (due to unanticipated challenges).

^Implementation Support Strategies 1.1-7.3 are outlined in Sutherland et al 2020 (6).

**PA4E1 Implementation Team includes the Program Manager, Individual Practitioners (Support Officers from all four Local Health Districts, as well as additional Project Staff).

Abbreviations: N/A = Not applicable; PE: physical education; PA4E1 = Physical Activity 4 Everyone; AUD = Australian Dollars

**References**

1. Sutherland R, Campbell E, Nathan N, Wolfenden L, Lubans DR, Morgan PJ, et al. A cluster randomised trial of an intervention to increase the implementation of physical activity practices in secondary schools: study protocol for scaling up the Physical Activity 4 Everyone (PA4E1) program. BMC Public Health. 2019;19(1):883.

2. Stirman SW, Miller CJ, Toder K, Calloway A. Development of a framework and coding system for modifications and adaptations of evidence-based interventions. Implementation Science. 2013;8(1):65.

3. Rabin BA, McCreight M, Battaglia C, Ayele R, Burke RE, Hess PL, et al. Systematic, Multimethod Assessment of Adaptations Across Four Diverse Health Systems Interventions. Frontiers in Public Health. 2018;6(102).

4. Wiltsey Stirman S, Baumann AA, Miller CJ. The FRAME: an expanded framework for reporting adaptations and modifications to evidence-based interventions. Implementation Science. 2019;14(1):58.

5. Kirk MA, Moore JE, Wiltsey Stirman S, Birken SA. Towards a comprehensive model for understanding adaptations’ impact: the model for adaptation design and impact (MADI). Implementation Science. 2020;15(1):56.

6. Sutherland R, Campbell E, McLaughlin M, Nathan N, Wolfenden L, Lubans DR, et al. Scale-up of the Physical Activity 4 Everyone (PA4E1) intervention in secondary schools: 12-month implementation outcomes from a cluster randomized controlled trial. International Journal of Behavioral Nutrition and Physical Activity. 2020;17(1):100.
